# Chronic Cough in Adults: Make the Diagnosis and Make a Difference

**DOI:** 10.1007/s41030-019-0089-7

**Published:** 2019-03-13

**Authors:** Alan G. Kaplan

**Affiliations:** University of Toronto, Toronto, Canada

**Keywords:** Adult, Cough, Spirometry

## Abstract

Chronic cough is common and impactful, frustrating both patients and clinicians. An empirical trial of therapy is often done with inhaled corticosteroids, but this practice should be replaced with attempting to make an accurate diagnosis. The three most common causes are upper airway cough syndrome, asthma, and gastroesophageal reflux disease (GERD), but there are often multiple causes involved. Minimal investigations after history, physical exam, travel history, and drug history include a chest radiograph and spirometry. Empirical trial of therapy with inhaled corticosteroids is reasonable if there is evidence of eosinophilic inflammation. Empiric therapy for GERD may also be reasonable in those with symptoms. Red flags should especially be considered an urgency to make the correct diagnosis.

## Introduction

The management of chronic cough presents a challenge for the clinician. Chronic cough is typically defined as a cough that persists for longer than 8 weeks. Interestingly, it is the most common symptom presenting in adults who seek medical treatment in non-hospital settings [1, 2]. Chronic cough can occur in up to 40% of the population [3]. Unexplained chronic cough remains the answer is up to 10% of cough patients, occurring in up to 46% of patients referred to specialty cough clinics [4]. Empirical therapy is commonly done, which may explain why success rates are suboptimal.

Chronic cough can arise from anywhere in the tracheobronchial tree, and as such may require a multidisciplinary approach. The primary care provider will often need to coordinate the care with referrals to any of the specialties including otolaryngologist, pulmonologist, gastroenterologist, allergist and immunologist, neurologist, and speech therapy, depending on the presumed diagnosis. The most common causes of chronic cough are UACS, asthma, GERD, nonasthmatic eosinophilic bronchitis, or importantly some combinations of these four conditions, but the differential continues to be quite broad. One should also consider medication side effects and of course the red flags. This review shall provide an approach to the assessment, diagnoses, and potential management strategies while emphasizing the need to be as specific with a diagnosis as possible for proper outcomes.

### Definitions of Acute, Subacute, and Chronic Cough

Using the American College of Chest Physicians [5] most recent guidelines, cough is divided into acute, subacute, and chronic cough. For adult patients complaining of cough, they suggest that acute cough be defined as being < 3 weeks in duration, subacute cough be defined as being between 3 and 8 weeks in duration, and that chronic cough be defined as being > 8 weeks in duration. Etiology should be considered based both on duration and location around the globe. This review will focus on chronic cough.

## Mechanism of Cough

Cough is a normal physiologic process that involves a protective reflex to allow clearing of debris and secretions from the lungs and airways. It contains three components: an afferent sensory limb, a central processing center, and an efferent limb [6].

The afferent pathways have cough receptors supplied by the trigeminal, glossopharyngeal, and vagus nerves. The vagus nerve supplies most of these receptors through the pharyngeal, superior laryngeal, and pulmonary branches. There are cough receptors are within the airways from the pharynx to the terminal bronchioles, with the largest concentration present in the larynx, carina, and the bifurcation of larger bronchi [7]. The receptors respond to different stimuli including mechanical stimuli, pulmonary congestion, atelectasis, bronchoconstriction, cigarette smoke, ammonia, acidic and alkaline solutions, hypotonic and hypertonic saline, histamine, bradykinin, prostaglandins, substance P, and capsaicin.

The impulses from the afferent nerves transmit to the cough center of the brain, located in the nucleus tractus solitarius of the medulla of the brainstem. This stimulates the central respiratory generator. The reflex arc is completed when impulses are sent via the vagus nerve and supply the phrenic and spinal motor nerves of C3 to S2 supply the intercostals muscles, abdominal wall, diaphragm, and pelvic floor, which assist the generation of the cough.

One of the key factors in understanding how chronic cough may no longer respond to respiratory treatments is that the cough reflex has been shown to have neuroplasticity. The definition of neuroplasticity [8] is the ability of the brain to form and reorganize synaptic connections, especially in response to learning or experience or following injury. With repeated stimulation, the cough induces chronic irritation and inflammation in the tissues and the nerves supplying them. This leads to remodeling, which leads to the tissues and nerves becoming sensitized [9]. This sensitization occurs both peripherally by increasing the sensitivity of cough receptors, and centrally, by actually changing processing in the brainstem, which accounts for the exaggerated cough response. This also contributes to the continuation of chronic cough [10]. This is akin to how chronic neuropathic pain develops and the relevance of this will be reviewed in the treatment section.

## Diagnostic Tools

The first step in making a diagnosis is the history. This should include duration and progression of cough, associated or systemic symptoms such as fever, chills or weight loss, hemoptysis, travel history, current medications, and effective vs. ineffective treatments trialed. Contrary to what one might think, the patient’s description of the type of cough, when it occurs, and whether there is associated sputum has been shown to have little diagnostic value [11, 12]. The medical history is important to ascertain whether the patient is or has been a smoker; is taking an ACE (angiotensin converting enzyme) inhibitor; is living in a geographic area endemic for TB or certain fungal diseases, has any systemic symptoms, or a history of cancer, tuberculosis, or AIDS (Acquired Immunodeficiency Syndrome). Specific next steps would depend on diagnostic considerations.

Recurrent aspiration can only be diagnosed by a good history and firstly actually considering the diagnosis. Subsequently, one must either watch the patient drink water or involve a speech-language pathologist to do so.

Pertussis is more commonly a childhood illness with a catarrhal stage of mild cough, nasal congestion, and fever lasting about 3 weeks, which then transforms into the paroxysmal cough that can last months. It is infectious mostly in the first 2 weeks when the symptoms are less specific. A high index of suspicion is required to consider this. The diagnostic test is a nasopharyngeal swab with a special medium as the organism is quite difficult to grow. In adults, the diagnostic “whoop” is usually absent, but the thick tenacious mucus and severity of the cough that may be associated with syncope or vomiting can be a consideration.

Post nasal drip syndrome (PNDS) is the most common cause of upper airway cough syndrome (UACS). It is described as the feeling of secretions draining from the nose or sinuses into the pharynx and is often associated with nasal congestion or discharge or frequent throat clearing. This is largely based on a patient’s subjective symptoms and may not show any significant physical examination findings. In addition, up to 20% of patients with PNDS-induced cough are unaware of the presence of postnasal drip or its link to their cough [13]. Physical findings such as the presence of mucus in the oropharynx or cobblestoning of the oropharyngeal mucosa may suggest the diagnosis, but while they are sensitive, they are not specific [14]. A nasal speculum examination is often underperformed and can reveal congestion, septal deviation, or nasal polyps.

**Fig. 1:**
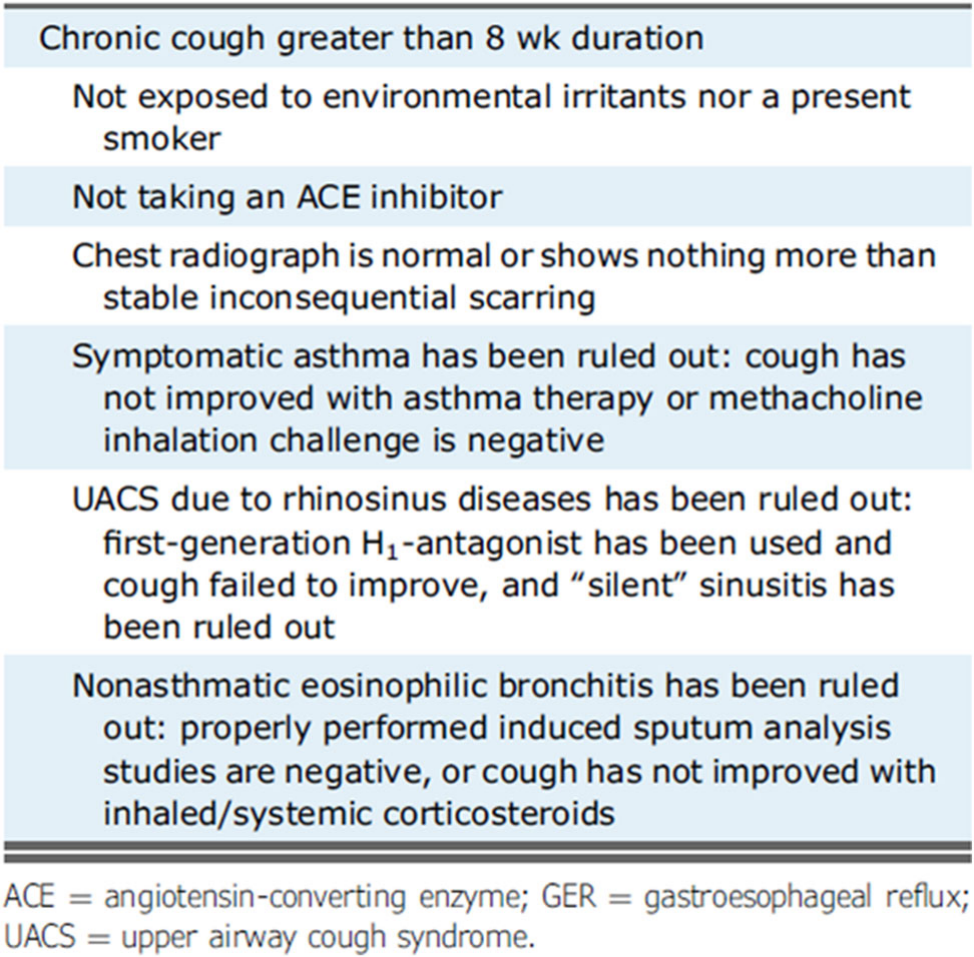
Consideration for asthma and non-asthmatic eosinophilic bronchitis should also be considered prior to empirical treatment for GERD. Reprinted from CHEST, 150/6, Peter J. Kahrilas, Kenneth W. Altman, Anne B. Chang, Stephen K. Field, Susan M. Harding, Andrew P. Lane, Kaiser Lim, Lorcan McGarvey, Jaclyn Smith, Richard S. Irwin, Todd M. Adams, Kenneth W. Altman, Elie Azoulay, Alan F. Barker, Fiona Blackhall, Donald C. Bolser et al. Chronic cough due to gastroesophageal reflux in Adults CHEST Guideline and Expert Panel Report, 1341–1360, Copyright (2016), with permission from Elsevier

There is a 92% probability, based on prospective trials, that in patients who have been treated unsuccessfully for UACS, asthma, and NAEB, not on an ACE and with a normal CXR, that their chronic cough is due to GERD [11]. Classic symptoms such as reflux, heartburn, and less classic such as dental erosions or voice changes should be considered but may not be present. Consideration for asthma and non-asthmatic eosinophilic bronchitis should also be considered prior to empirical treatment for GERD [15] (Fig. 1).

Spirometry is the gold standard to diagnose asthma and COPD, but false positives can exist with some conditions that can cause confusion. Bronchiectasis can certainly have some reversible obstruction at some stages of the illness and its clinical hallmark is productive cough. Restriction, not obstruction, is the hallmark of other underlying lung diseases such as interstitial pulmonary diseases [16], which could be secondary to connective tissue diseases, medications, or idiopathic. Cough and dyspnea are the most common presentations, and clinical exam may show inspiratory crackles, often of a Velcro-like sound, which can be helpful.

An abnormal chest radiograph will define the further workup needed, depending on the specific findings. Chest CT scan, bronchoscopy, needle biopsy, and sputum studies are all potential next steps if a pulmonary lesion is found.

Fractional exhaled nitric oxide (FENO) can be of value to consider which patients would respond to inhaled corticosteroids (ICS). In a study of asthmatic patients in whom 85% had cough, ICS showed tremendous benefits in patient improvement if FENO > 50 ppb vs. those with FENO < 50 ppb [17]. In a similar Chinese study, FENO also correlated nicely with improvement in patients treated with ICS and this population was patients with chronic cough and normal CXR [18]. In a British study of patients collected from a cough clinic, there was a significant correlation between FENO, blood eosinophils, and sputum eosinophil count (*p *< 0.001), which again shows that FENO can predict a more likely ICS responsive group in patients presenting with only cough [19].

**Fig. 2:**
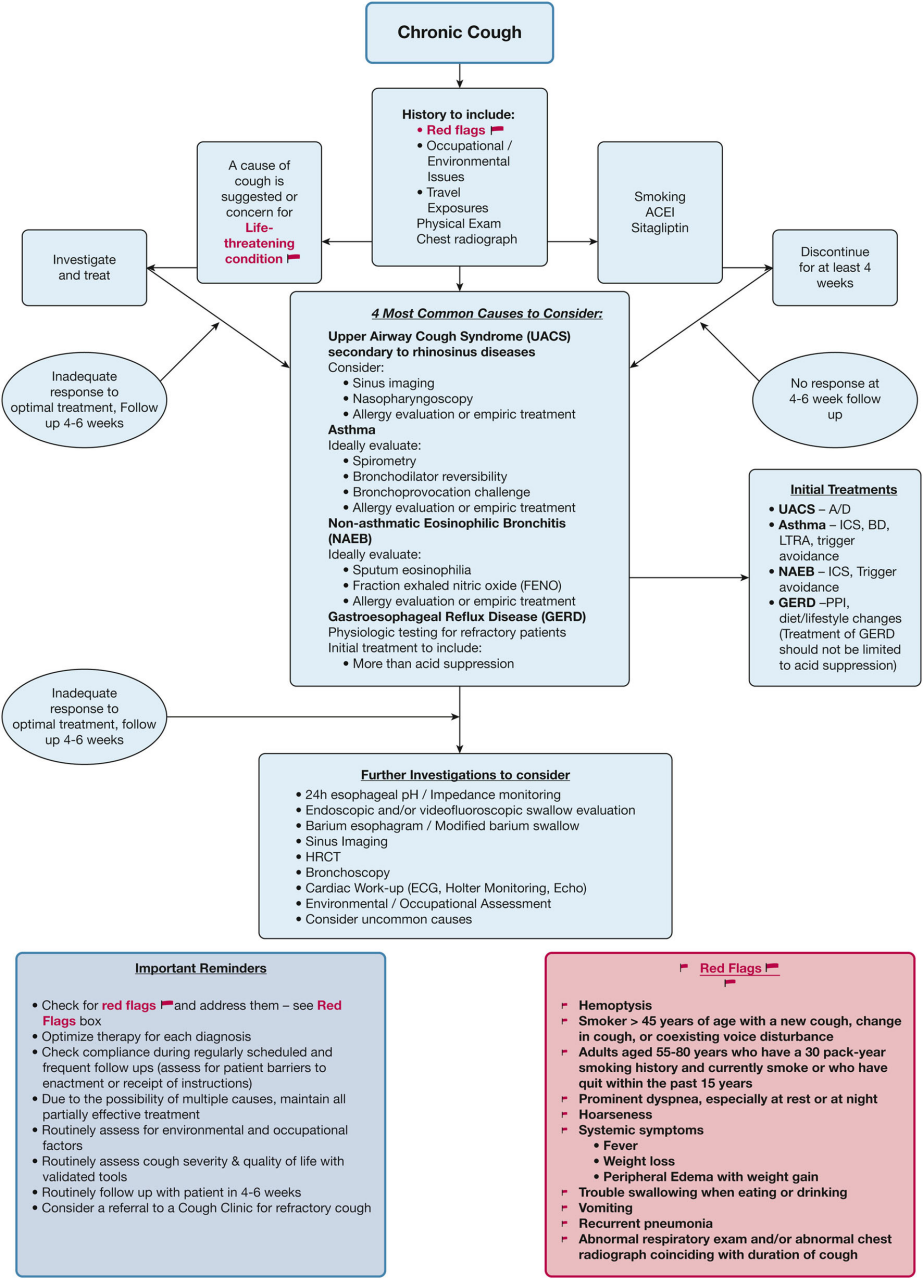
Overview of the diagnostic approach. Reprinted from CHEST, 153/1, Author(s), Richard S. Irwin, Cynthia L. French, Anne B. Chang, Kenneth W. Altman, Todd M. Adams, Kenneth W. Altman, Elie Azoulay, Alan F. Barker, Surinder S. Birring, Fiona Blackhall, Donald C. Bolser, Louis-Philippe Boulet, Christopher Brightling, Priscilla Callahan-Lyon et al. 196–209, Copyright (2018), with permission from Elsevier

The following algorithm gives an overview of the diagnostic approach (Fig. 6 from ACCP guidelines) (Fig. 2).

## Management

Attempts at looking for a one-size-fits all approach are unfortunately doomed to failure. A meta-analysis of treatments for subacute cough found no evidence of benefit with montelukast, salbutamol, fluticasone propionate, budesonide, opioids, or codeine [20].

One straightforward way to distinguish the cause and subsequent management of the cough is to divide it up between eosinophilic airway diseases (asthma and NAEB) and noneosinophilic chronic cough [21]. Eosinophilic airway diseases can be diagnosed by raised induced sputum eosinophil counts and increased fractionated exhaled nitric oxide levels, and perhaps suspected with elevated blood eosinophils. These patients will more likely respond well to corticosteroids, but otherwise the routine use of ICS for cough should be discouraged without an attempt being made to discover the etiology and treat as specifically as possible [22].

The first steps in managing chronic cough should be stopping smoking or the use of an ACE inhibitor if these are occurring. This will lead to a resolution of the cough within 4 weeks of smoking cessation, albeit the cough can remain lifelong [16] and cough related to ACE inhibitor use usually subsides within 2 weeks, although the median time has been reported to be 26 days (16). The mechanism of ACE inhibitor-induced cough is not completely clear, but likely involves the protussive mediators bradykinin and substance P. These chemicals are usually degraded by ACE, and as such will accumulate in the upper respiratory tract or lung when the enzyme is inhibited [23].

Prostaglandins are stimulated by bradykinin and may contribute to the cough. There have even been some cases of ARB (angiotensin receptor blocker)-induced cough, although bradykinin accumulation should not occur with ARBs [24].

Sitagliptin has been reported to have a syndrome including cough, dyspnea, fatigue, and rhinorrhea, interestingly more often in patients with ACE-induced cough and/or a history of allergic rhinitis. Rhinorrhea, cough, and fatigue generally improved in the first week off sitagliptin, while PEFR took 1–3 weeks to improve [25].

In immunocompetent non-smoking patients who are not using ACE inhibitors with a normal chest X-ray (CXR), the best approach is to use a systematic approach tailored to the most common causes of chronic cough. Frankly, the accuracy of the diagnosis is confirmed by the patient’s response to these treatments. A fairly quick initial work-up therefore includes history, physical exam, medication history, CXR, and spirometry. As such, from both theoretical and cost-effectiveness standpoints, starting with empiric treatment of the three most common causes of cough (UACS, asthma, GERD) is favored over extensive testing [26, 27]. Additional therapy, not just changing the therapy, may be needed, as there may be more than one cause of the cough.

### Big Three

#### Upper Airway Cough Syndrome

Because upper airway cough syndrome (UACS) is the most common cause of chronic cough, it should be specifically treated first. Potential treatment includes avoiding environmental irritants and offending antigens, treating sinusitis with saline lavage, nasal inhaled corticosteroids, antihistamines, and antibiotics. Remember to enquire about and subsequently wean patients off nasal decongestants for rhinitis medicamentosa. Further work-up may include allergy testing for allergic rhinitis or sinus CT scan for sinusitis, as indicated. Patients typically respond to UACS therapy within 2 weeks but this can take several months [13].

#### Asthma

Asthma should be considered only after the UACS evaluation and empirical treatment trial are complete. Ideally, patients should undergo spirometry and consideration could be made if normal for bronchoprovocation challenge with methacholine. A negative methacholine challenge at the time of symptoms rules out asthma virtually all of the time.

Asthma management includes trigger avoidance, smoking cessation, anti-inflammatory therapy with ICS or potentially leukotriene inhibitors. Bronchodilators include beta agonists, which should be given in the company of ICS. Tiotropium on top of ICS or ICS/LABA, a long-acting antimuscarinic, may also help improve lung function, reduce symptoms, and decrease exacerbations [28].

COPD more likely presents with dyspnea, but chronic bronchitis can be associated with long-term cough associated with such.

#### Gastroesophageal Reflux Disease

The diagnosis of GERD can be critically performed by the use of dual-channel 24-h pH probe monitoring, but this is not a first-line investigation and should be considered in those refractory to therapy. In those with any voice changes, flexible nasopharyngoscopy can reveal changes in the glottis that are known to occur with exposure to the products of reflux. These include laryngeal edema and erythema, laryngeal pseudosulcus, and posterior commissure hypertrophy or pachydermia.

Empiric treatment with lifestyle and dietary modification in addition to acid suppression therapy is likely preferred initially instead of testing, which should be reserved for refractory cases. Lifestyle modifications include diet modification include the promotion of weight loss in overweight or obese patients, elevation of the head of the bed while sleeping, avoiding meals within 3 h of bedtime, limiting fat intake, avoiding substances that lower the lower esophageal sphincter pressure such as caffeine, chocolate, mints, citrus products, alcohol, and smoking, and limiting vigorous exercise that increases intra-abdominal pressure [29].

While many acid-suppressive therapies are available including histamine 2 receptor blockers, proton pump inhibitors (PPIs), sulcrafate and antacids, the maximal medical therapy refers to twice-daily PPI in addition to a prokinetic agent along with lifestyle and dietary modifications. Response can be seen in as little as 2 weeks, and at least a 6- to 8-week trial is needed to fully evaluate a response to treatment, with some patients requiring as long as 6 months [30, 31]. If an adult patient does not actually have any symptoms of heartburn or regurgitation, PPI therapy empirically alone is unlikely to be effective in resolving the cough [15]. Side effects of long-term PPIs include hypomagnesemia, increased risk of hospital-acquired pneumonia, and of* Clostridium difficile* infections, fractures, and B12 deficiency. Side effects of currently available prokinetic agents include (with domperidone) prolonged QT interval, sexual side effects and galactorrhea among others, while with metoclopramide they include tardive dyskinesia, tremors, and sedation among others.

#### Nonallergic Eosinophilic Bronchitis

Nonallergic eosinophilic bronchitis (NAEB) can be diagnosed with an induced sputum test showing airway eosinophilia in the context of normal airway function testing, bronchoscopy with lavage, and potentially biopsy. Treatment includes ICS, with oral corticosteroids not uncommonly needed for refractory cases. Response is usually seen within 4 weeks [15].

## Refractory Cough

Ensure that the cough lasting > 8 weeks had a work-up that was completed and therapy that was adhered to. Then, consider objective testing for bronchial hyperresponsiveness and eosinophilic bronchitis, or a therapeutic corticosteroid trial. In the absence of such, although it is commonly done and advised, therapy with ICS should not be prescribed. If still unsuccessful, consider a therapeutic trial of multimodality speech pathology therapy.

**Fig. 3:**
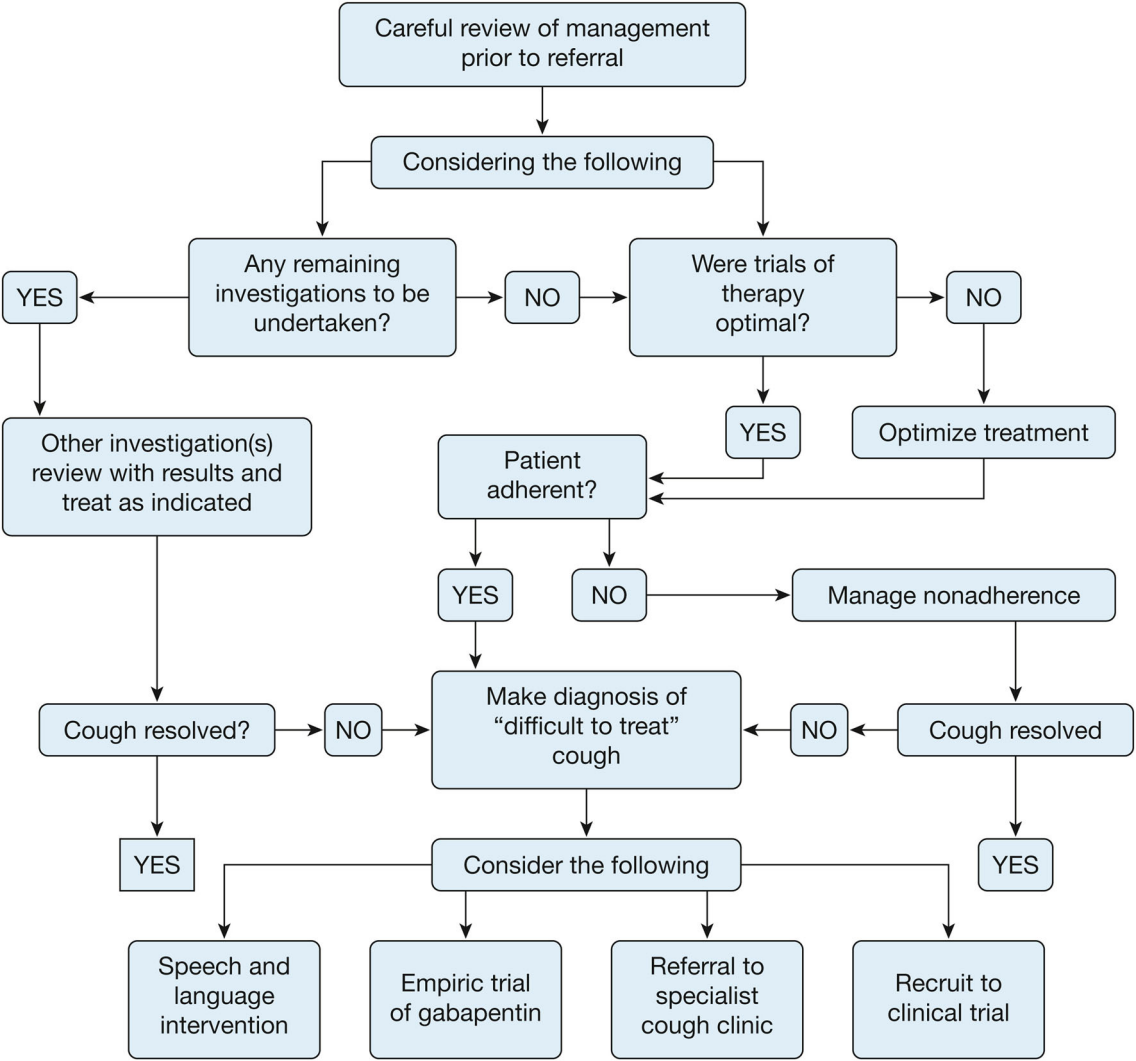
Algorithm detailing a management approach for “difficult-to-treat” cough. Reprinted from CHEST, 149/1, Peter Gibson, Gang Wang, Lorcan McGarvey, Anne E. Vertigan, Kenneth W. Altman, Surinder S. Birring, Todd M. Adams, Kenneth W. Altman, Alan F. Barker, Surinder S. Birring, Fiona Blackhall, Donald C. Bolser, Louis-Philippe Boulet, Sidney S. Braman et al. Treatment of Unexplained Chronic Cough CHEST Guideline and Expert Panel Report, 27–44, Copyright (2016), with permission from Elsevier

If even further testing such as sputum for AFB or bronchoscopy (depending on presentation) or even a referral to a speech and language pathologist does not reveal the cause [32], then the patient most likely has chronic cough hypersensitivity syndrome. Neuroplasticity occurs because of the inflammation and hyperresponsiveness of the airway from the inciting causes that are not resolved, subsequent tissue remodeling and nerve sensitization, all of which lead to an enhanced cough reflex that maintains the cough even though the inciting cause has resolved [33]. Similarities have been demonstrated between neuropathic pain and chronic cough, and centrally acting neuromodulators such as tricyclic antidepressants (amitriptyline, nortriptyline), gabapentin, and pregabalin have shown benefit in improving cough (albeit with risk of side effects) [34–36]. Consider the potential side effects and the risk–benefit profile of these medications and again at 6 months before continuing the drug (Fig. 3).

### Cough Hypersensitivity Syndrome (CHS)

The cough hypersensitivity syndrome had been considered in 2010 [37] and was endorsed as a syndrome by ERS in a task force begun in 2011. The concept of hypersensitivity of airway sensory nerves leading to cough has further been expanded and investigated [38]. They felt that cough sensitivity is distinct from methacholine bronchial responsiveness and reflects afferent perception of a wide and perhaps different range of stimuli. They felt that CHS, defined as “a clinical syndrome characterized by troublesome coughing often triggered by low levels of thermal, mechanical or chemical exposure”. Interestingly, this definition of CHS is purely clinical and does not require any formal testing to demonstrate hypersensitivity of the cough reflex (which is completely distinct from bronchial hyperresponsiveness (BHR) to e.g., methacholine). Possible triggers include exposure to environmental tobacco smoke, spicy food, perfumes, vapors, changes in ambient temperature, and others.

There have been a few different purported tests for this condition including the capsaicin cough threshold test [39]. Unfortunately, thought there are currently available tests using capsaicin or citric acid to explore the sensitivity of afferent pathways they are not specific to well-defined abnormalities/mechanisms or useful from a diagnostic perspective at an individual level [40]. As such, the results of these tests cannot be used to distinguish patients with from those without CHS, nor CHS patients with from those without underlying diseases such as asthma, COPD, or GERD [41].

The use of local anesthetics either orally or inhaled has also been investigated somewhat. An oral formulation called benzonatate is thought to act as a local anesthetic, decreasing the sensitivity of stretch receptors in the lower airway and lung, thereby reducing the drive to cough after taking a deep breath. It is not benign, with side effects including drowsiness, dizziness, dysphagia, and pulmonary aspiration (if the mouth is numb from the anesthetic). There is also concern that if the gelcaps are chewed or allowed to dissolve in the mouth, it could lead to an overdose of the drug, which would present with central nervous system side effects, such as mental confusion and hallucination, restlessness, and tremors, potentially even with convulsions and death [42].

A much safer alternative is to inhale lidocaine. This can be done safely by either nebulizing it [43] or, where available, using a lidocaine throat spray, which may be even more efficacious [44].

The effectiveness of medications working on neuropathic mechanisms, such as gabapentin and pregabalin, in the treatment of refractory cough has been supported primarily through case series, case reports, prospective reviews, and a double-blind randomized controlled trial. Gabapentin results in a reduction in cough frequency and cough severity. It improved questionnaire-based measurements of cough (Leicester Cough Questionnaire) as well as objective measurements of capsaicin sensitivity. It also improves cough related quality of life [45]. Side effects of gabapentin include somnolence and dizziness [46].

Opioid therapy systemically has been suggested in refractory cases and could be considered when etiologies such as lung cancer or pulmonary fibrosis occur to also assist in terminal breathlessness [47, 48]. That being said, the use of opioid cough suppressants should be done with extreme caution due to their lack of efficacy and risk of abuse. In the United States, they have been combined with soft drinks and have names like “Lean and Purple” [49].

This is not a small problem; in 2014, the United States Drug Enforcement Agency believed that one in ten teens abused codeine cough syrups to get high. The Substance Abuse and Mental Health Services Administration reported in 2008 that three million adolescents and young adults, usually between the ages of 12 and 25, had used cough or cold medicines to get high; this number included those using prescription-only codeine-based cough syrup and over-the-counter dextromethorphan drugs. When recommended doses are exceeded and if mixed with other products, a severe dissociative, “out-of-body” experience can occur, similar to the effects of well-known hallucinogens such as phencyclidine (PCP) and ketamine (“Special K”) [50].

Table of potential drug therapies not shotgun, but targeted based on history, physical and investigations.

*Eosinophilic disease* ICS/OCS.

Asthma, also include LABA, LAMA, LTRA, or biologics.

*COPD* Bronchodilators.

*Bronchiectasis* Treat potentially the underlying infection, e.g., TB, atypical TB.

Interstitial lung diseases, treat or remove the causation; if IPF, consider specific therapies.

*Upper Airway cough syndrome* Nasal lavage and nasal ICS.

*GERD* PPIs, prokinetics.

*CHS* Gabapentin, prebaglin, lidocaine.

## Conclusions

Chronic cough is common, disabling, and may not have a readily obvious cause. The use of an algorithmic approach such as the ACCP guidelines can give you a roadmap to making the diagnosis. It is tempting to just try ICS, but in the absence of evidence of eosinophilic inflammation, this should be avoided. Go to the effort of making a firm diagnosis, most often one of the big three of UACS, GERD, or asthma, especially in those without any ‘red flags’ and your success of treatment will be much higher.
